# Anorexia of Aging: A Modifiable Risk Factor for Frailty

**DOI:** 10.3390/nu5104126

**Published:** 2013-10-14

**Authors:** Anna Maria Martone, Graziano Onder, Davide Liborio Vetrano, Elena Ortolani, Matteo Tosato, Emanuele Marzetti, Francesco Landi

**Affiliations:** Department of Geriatrics, Neurosciences and Orthopedics, Teaching Hospital “Agostino Gemelli”, Catholic University of Sacred Heart, Rome 00168, Italy; E-Mails: annamariamartone@gmail.com (A.M.M.); graziano.onder@rm.unicatt.it (G.O.); davidevetrano@gmail.com (D.L.V.); ele.ort@gmail.com (E.O.); matteo.tosato@rm.unicatt.it (M.T.); emarzetti@live.com (E.M.)

**Keywords:** elderly, sarcopenia, ghrelin, malnutrition, weight loss, disability, energy metabolism

## Abstract

Anorexia of aging, defined as a loss of appetite and/or reduced food intake, affects a significant number of elderly people and is far more prevalent among frail individuals. Anorexia recognizes a multifactorial origin characterized by various combinations of medical, environmental and social factors. Given the interconnection between weight loss, sarcopenia and frailty, anorexia is a powerful, independent predictor of poor quality of life, morbidity and mortality in older persons. One of the most important goals in the management of older, frail people is to optimize their nutritional status. To achieve this objective it is important to identify subjects at risk of anorexia and to provide multi-stimulus interventions that ensure an adequate amount of food to limit and/or reverse weight loss and functional decline. Here, we provide a brief overview on the relevance of anorexia in the context of sarcopenia and frailty. Major pathways supposedly involved in the pathogenesis of anorexia are also illustrated. Finally, the importance of treating anorexia to achieve health benefits in frail elders is highlighted.

## 1. Introduction

Accumulating evidence indicates that a significant number of frail elderly people fail to ingest an amount of food that meets essential energy and nutrients needs [1]. Anorexia of aging and consequent weight loss are very common problems among the elderly, especially in nursing home residents and in hospitalized older patients [1,2,3]. Anorexia is defined as a loss of appetite and/or reduced food intake. Anorexia is a true geriatric syndrome because it is a multifactorial condition associated with multiple negative health outcomes [4]. It generally develops when the accumulated effects of impairments in multiple systems make the older subject more vulnerable to adverse health events.

Given this definition, it is readily understandable why anorexia and frailty are strongly interconnected and can influence one another. Studies have shown that anorexia is highly prevalent among older adults, affecting over 25% and 30% of elderly men and women, respectively [1,2].

## 2. Risk Factors for Anorexia of Aging

The pathogenesis of anorexia is thought to involve age-related declines in the activities of specific brain areas, including the hypothalamus, in response to peripheral stimuli (fat cell signals, nutrients, circulating hormones) [5,6,7]. Inflammation, which is linked to the aging process, contributes to the pathogenesis of anorexia and also plays a key role in anorexia associated with chronic diseases or cachexia [7,8].

As described by Serra-Prat and colleagues [9], older frail persons exhibit an impaired response to hunger hormones such as ghrelin and cholecystokinin (CCK). Furthermore, a 40% fat meal increases glucagon-like peptide 1 (GLP-1) levels and decreases the acylated-to-desacylated ghrelin ratio in elderly, but not in young adults [5]. These alterations may be responsible for decreased hunger and unintended weight loss in old age. Similarly, the infusion of low doses of CCK causes a reduction in calorie intake in the elderly, but not in young people. It has also been suggested that levels of GLP-1 and ghrelin increase insulin sensitivity in certain brain areas such as the hypothalamus, causing a dysregulated energy homeostasis, which leads to reduced food intake [5]. Furthermore, pro-inflammatory cytokines persistently activate pro-opiomelanocortin neurons and inhibits neuropeptide Y neurons involving an alteration in satiety and hunger signals, the clinical expression of which is represented by anorexia and cachexia [8,10]. Other age-associated conditions, such as alterations in taste and smell [11], decreased chewing efficiency often due to edentulism [12], delayed gastric emptying [13], spontaneous gastroesophageal reflux [14], declines in gastric and pancreatic exocrine secretions [15,16], reduced testosterone levels in men leading to elevated leptin levels [17], and depression [18], also contribute to the pathogenesis of anorexia of aging. In addition to a global reduction in food intake, elderly individuals with anorexia also exhibit altered eating patterns, characterized by lower consumption of nutrient-rich foods (e.g., meat, eggs and fish) [19].

Anorexia should not be considered as an inevitable “side effect” of aging; rather, many risk factors can be identified and potentially amended. They are represented by the aging process itself, changes in body composition (such as the reduction in fat-free and muscle mass and increase in visceral fat), functional impairment, social and environmental conditions, acute and chronic diseases and their treatments [20,21].

**Figure 1:**
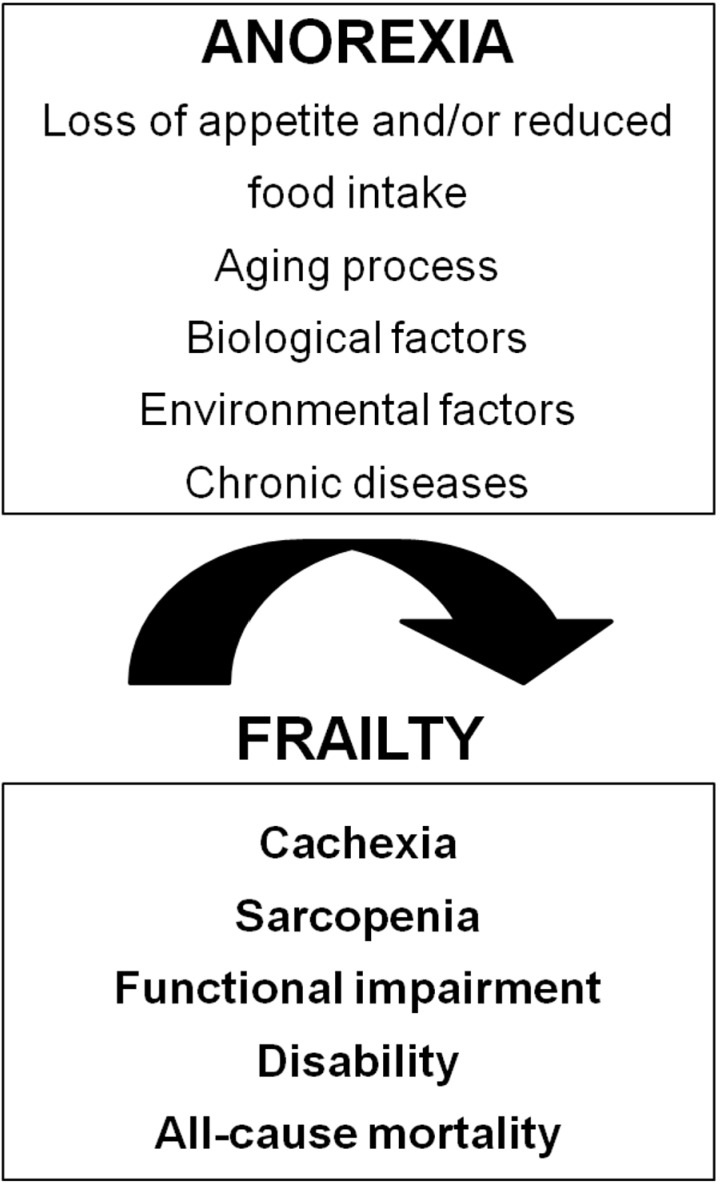
Anorexia as a geriatric syndrome.

It is important to highlight that potentially reversible causes are strongly associated with the onset of anorexia during the aging process [22,23]. Functional, psychological and economical factors contribute to secondary anorexia and malnutrition. For instance, functional impairments in basic and instrumental activities of daily living, loneliness, lack of cooking skills, depression, economic concerns, weakness, and fatigue play a significant role in the development of anorexia. These elements—together with the physiological changes associated with aging—contribute to reducing food intake to levels lower than those required to meet energy and nutrient demand (Figure 1).

## 3. Consequences of Anorexia of Aging

Weight loss due to anorexia contributes to muscle wasting and decreased function of respiratory muscles, impaired immune function with depressed antibody- and cell-mediated responses and consequent greater susceptibility to infections, reduced gut function and increased bacterial translocation [24]. Furthermore, anorexia induces hypoalbuminemia, increased synthesis of acute-phase proteins such as C-reactive protein, α-1 acid glycoprotein and fibrinogen, and decreased coagulation capacity leading to oxidative stress and enhanced tissue damage [8,24].

In the early stage of anorexia, these effects could be attributed to low intake of single nutrients, in particular protein and certain vitamins [25]. This selective malnutrition is also directly correlated with the onset of sarcopenia [26]. This could explain why in most cases anorexia is associated with poor endurance, slow gait speed and decreased mobility [27]. However, the most important evidence from the current literature is that anorexia is an independent and strong predictor of morbidity and mortality among patients in various clinical settings [28].

## 4. Correlation among Anorexia, Sarcopenia and Frailty Syndrome

**Figure 2:**
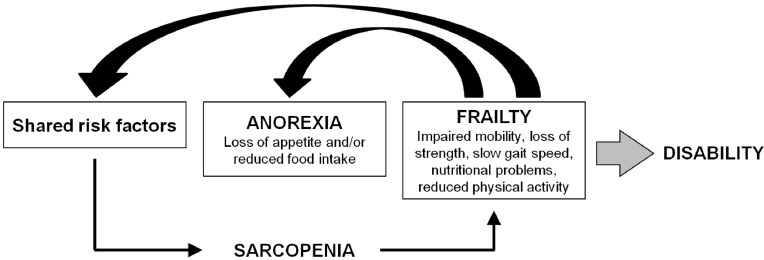
The interaction among anorexia of aging, sarcopenia and frailty.

Frailty is a geriatric syndrome characterized by a reduction of the physiological functional reserve and a decreased homeostatic capacity leading to greater vulnerability to adverse health outcomes and increased risk of death (Figure 2) [29].

Fried *et al.* [29] characterized the clinical phenotype of frailty and suggested an “operational definition” based on the identification of five parameters: (1) low handgrip (<5.85 kg in males; <3.37 kg in females); (2) self-reported fatigue (at least 3 times a week); (3) unintended weight loss (≥4.5 kg in the last year); (4) reduction of physical activity (as assessed by the PASE-Physical Activity Scale for Elderly); and (5) slow walking velocity (≥7 s to walk a 4.57 m course). According to the Fried’s criteria, older persons are defined frail if they meet at least three parameters.

The decrease in food intake associated with anorexia leads to a reduction in exercise capacity and declines in muscle mass and strength [25], and is therefore involved in the development of the frailty syndrome. Furthermore, as suggested by Fried *et al.* [29], sarcopenia plays an important etiologic role in frailty, being also a key player of its latent phase and explaining many aspects of the frailty status itself.

In a recent study, members of our group evaluated the relationship between anorexia and sarcopenia in 354 community-dwelling persons aged 80 years [26]. A direct association was found between them: 34 (46.6%) subjects were affected by sarcopenia among those with anorexia, compared to 69 subjects (24.6%) without anorexia. The correlation was independent of overt malnutrition and remained significant after adjusting for potential confounders such as age, sex, functional and cognitive impairment, level of physical activity, comorbidity, depression, anticholinergic drug use and tumor necrosis factor-α (TNF-α) plasma levels. These results are consistent with the shared etiopathogenetic mechanisms mentioned above.

Recent studies have suggested that supplementation with essential amino acids promotes muscle growth by improving muscle insulin sensitivity through increasing insulin-like growth factor (IGF)-signaling and down-regulating pro-inflammatory cytokines, such as TNF-α [30,31]. In particular, leucine, which is abundant in whey, is a secretagogue of insulin and a powerful activator of the mammalian target of rapamycin (mTOR), the master regulator of protein synthesis [31].

The current recommended dietary allowance (RDA) for protein is 0.8 g/kg/day. However, older people do not usually ingest this amount. It is reported that 32%–41% of women and 22%–38% of men aged ≥50 years consume less than the RDA for protein [32]. Furthermore, older adults show a decreased anabolic response to protein meals, implying that the current RDA may not protect elderly individuals from developing sarcopenia. In this perspective, many authors suggest that older people should increase their protein intake to 1.0–1.3 g/kg/day [33] and that this amount of protein should be consumed according to a spread pattern during the day in order to ensure an optimal muscular anabolic response [34].

In animal models, vitamin D deficiency and high parathyroid hormone (PTH) levels have been linked with higher muscleprotein breakdown [35]. Scott *et al.* [36] have demonstrated that serum levels of vitamin D are powerful, independent predictors of changes in muscle mass and strength over 2.6 years of follow-up. Further, vitamin D supplementation (800 IU daily) increases the number and cross-sectional area of type II fibers (which are typically lost in sarcopenic subjects) and that is associated with positive outcomes, including reduction of the risk of falls and fractures and improvements in muscle mass and strength [37].

Given these data, it is not surprising that physical function declines in the presence of anorexia, especially among those also experiencing weight loss. In this regard, we recently evaluated the relationship between anorexia and measures of physical performance, muscle strength and functional status in older community-dwellers [38]. All of the physical performance, muscle strength and functional measures showed significant associations with the presence of anorexia. These associations remained significant after adjustment for potential confounders. Besides, anorexia was associated with a higher risk of developing disability during two years of follow-up. These findings have been confirmed analyzing the impact of anorexia on one-year survival among 1904 participants enrolled in the ULISSE study, a project designed to evaluate the quality of care for older people living in an Italian nursing home [21].

## 5. Conclusions

Anorexia of aging represents one of the major challenges for geriatric medicine given its impact on quality of life, morbidity and mortality. Evidence indicates that decreased food intake is a robust predictor of mortality in frail elders. Anorexia should therefore be considered as a marker of profound perturbations of energy metabolism, prompting immediate clinical countermeasures. However, a more precise understanding of the mechanisms responsible for the age-associated alterations of energy metabolism is necessary to effectively treat/prevent anorexia of aging.

One of the most important goals in the management of older people is to optimize their nutritional status. To achieve this objective, the first step is to identify subjects at risk of anorexia by using second- and third-generation geriatric assessment tools. Care plans should also be implemented to ensure an adequate amount (and quality) of food to limit weight loss due to anorexia of aging. Multi-stimulus interventions may be required, including texture, flavor enhancement and feeding assistance. Finally, it is important to identify and eliminate all potentially reversible factors that promote anorexia during aging [21].
